# Simultaneous Quantification of Trace and Micro Phenolic Compounds by Liquid Chromatography Tandem-Mass Spectrometry

**DOI:** 10.3390/metabo11090589

**Published:** 2021-08-31

**Authors:** Dandan Wang, Liangxiao Zhang, Li Yu, Fei Ma, Peiwu Li

**Affiliations:** 1 Oil Crops Research Institute, Chinese Academy of Agricultural Sciences, Wuhan 430062, China; 7200201022@stu.jiangnan.edu.cn (D.W.); yuli01@caas.cn (L.Y.); mafei01@caas.cn (F.M.); peiwuli@oilcrops.cn (P.L.); 2 Key Laboratory of Biology and Genetic Improvement of Oil Crops, Ministry of Agriculture and Rural Affairs, Wuhan 430062, China; 3 Quality Inspection and Test Center for Oilseed Products, Ministry of Agriculture and Rural Affairs, Wuhan 430062, China; 4 Hubei Hongshan Laboratory, Wuhan 430070, China

**Keywords:** simultaneous quantification, isotopic ion, trace and micro-metabolites, LC-MS/MS

## Abstract

The simultaneous quantification of trace and micro metabolites is a bottleneck in food and biological analysis. Phenolic compounds are the most widely distributed and have various physiological functions. In this study, the strategy for the simultaneous liquid chromatography tandem-mass spectrometry (LC-MS/MS) quantification of 13 trace and micro phenolic compounds was proposed by taking product ions and isotopic ions as quantitative ions. The method validation results showed that the limits of detection (LODs) were from 0.01 to 9.84 μg/kg, and the limits of quantification (LOQs) were from 0.03 to 32.8 μg/kg. The intra-day precision and inter-day precision were below 8.4% and 14.4%, respectively. The recoveries ranged from 81.9% to 117.2%, and the matrix effects ranged from −11.5% to 13.7%, which indicated that the method has high sensitivity and suitable stability. The developed analytical method was applied to determine trace and micro constituents in rapeseed samples. The analysis results indicated that the contents of sinapine have significantly different between high and low total phenolic content rapeseeds. This method provides a reference strategy for the simultaneous quantitative analysis of other micro- and trace antioxidants.

## 1. Introduction

Phenolic compounds are an important kind of antioxidants with one or more than one phenolic hydroxyl group in their molecules, which play an important role not only in the physiological processes of plants themselves but also in human diseases and health. More importantly, phenolic compounds have important biological activities and pharmacological effects. They can lower blood sugar and blood fat levels; prevent cardiovascular and cerebrovascular diseases [1]; and have anti-inflammatory [2], antithrombotic [3], anti-cancer, and anti-mutagenic [4,5] effects. Moreover, polyphenols can increase immunity and reduce the risk of various diseases, such as atherosclerosis, diabetes, coronary heart disease, Alzheimer’s disease, and schizophrenia [6,7,8]. Current researches on the determination of phenols and metabolic pathways are moving toward high-throughput and high-resolution analysis [9]. However, it was found that the simultaneous analysis of high-content chlorogenic acid and other trace polyphenols from *Thymus* *serpyllum* L. herb was still impossible, although three different pretreatment methods, including maceration, heat-, and ultrasound-assisted, were employed [10]. Multiple phenolic compounds were identified and quantified, but the narrow linear range of polyphenols was all from 0.05 to 5 mg/mL [11], which is insufficient for trace and micro-metabolites together. The injection amount is currently changed to make high-content compounds or low-content compounds maintain a suitable and stable signal by diluting or concentrating the sample. For example, cucurbitaceous seed extracts were concentrated, and then 17 equivalent polyphenol compounds were detected [12]. However, we note that simultaneous quantitative analysis of metabolites with large differences in content still cannot be achieved. There are generally one or several high-content phenols and dozens of trace phenols in samples, such as sinapine in crucifers, isoflavone in soybean, and tyrosol in olive fruits. In addition, it is hard to simultaneously detect phenolic compounds in phenylpropanoid biosynthesis pathways because of the large content differences.

Edible vegetable oil is one of the main sources of phenolic compounds in people’s diets, according to the results of dietary intake assessments. Polyphenols have strong antioxidant physiological activity [13,14,15,16]. In recent years, various oil processing technologies have been optimized to improve the content of phenolic compounds in edible oil. Some processes can greatly increase the polyphenol content in edible oil, such as microwave radiation heating [17], high-temperature steam pretreatment [18], and dry fractionation processes [19]. The demand for high nutritional quality rapeseed oil is increasing with improvements in people’s quality of life.

Rapeseed is a raw material for rapeseed oil, and the determination and analysis of its nutritional quality formation mechanism are necessary and important for human health and the oil industry [20]. The total phenolic content (TPC) was determined using the Folin-Ciocalteau colorimetric method for 332 rapeseed samples collected from different producing areas as described [21]. The TPCs of rapeseeds from different producing areas are quite different in the previous research. To further analyze the differential phenolic compounds as well as the regulation mechanism of phenolic compounds in rapeseed, it is critical to establish a simultaneous detection technique for polyphenols. However, as the micro component, sinapine in rapeseed accounts for 65–85% of TPC, while other phenolic compounds are trace components. Due to the large differences in content, simultaneous detection cannot be achieved via conventional methods. Therefore, it is necessary to develop a simultaneous quantification method for antioxidants.

In recent years, isotopes have been widely used in quantitative analysis, including the commonly used isotope dilution mass spectrometry method. Isotope dilution mass spectrometry is often used in conjunction with gas chromatography, liquid chromatography, and inductively coupled plasma [22,23,24]. In isotope dilution mass spectrometry, the analyte is labeled with a stable isotope and used as an internal standard. The peak area ratio and the mass ratio of the internal standard and the analyte are determined by mass spectrometry to calculate the content of the analyte [25]. Isotope dilution mass spectrometry is an effective method to eliminate matrix effects in the quantification process. This approach can effectively correct system errors during sample pretreatment, ionization efficiency, and separation; thus, it can improve accuracy, precision, and reproducibility [26,27]. The method is widely used in the detection and analysis of pesticide residues [28,29,30], veterinary drug residues [31], natural toxins [32,33,34,35], heavy metal pollution [36], micronutrients [37,38], and vitamins [39,40,41]. However, the main factors restricting its development are the scarcity of isotope-labeled reagents and their high price. More importantly, the availability of multiple isotope-labeled target compounds is more difficult to achieve in targeted metabolomics analysis.

Here, we describe a new strategy using natural isotope quantification to reduce the difference in response among metabolites and achieve simultaneous detection of micro-components and trace components. This strategy solves the problem of simultaneous quantification of compounds with large differences in content. This approach not only achieves simultaneous quantification of trace and micro-metabolites but also improves the determination accuracy of multiple targeted compounds. The method has a significant promoting effect on the production of high-nutrient-quality rapeseed oil, the development of the rapeseed industry, and human nutrition and health.

## 2. Results and Discussion

### 2.1. Quantification by Isotopic Ion to Overcome Signal Saturation

The polyphenols in the rapeseed samples were extracted according to the extraction method and analyzed according to the optimized LC-MS/MS conditions. Sinapine is a quaternary ammonium salt, and thus, it was analyzed using positive ion scan mode ([M]^+^). However, high levels of sinapine in rapeseeds can lead to excessive signal response and excessive peak areas. Accurate quantification of sinapine and simultaneous analysis of multiple polyphenols cannot be achieved due to severe signal saturation. Signal saturation is still common when the quantitative analysis was attempted via ion fragments at low relative abundance. Currently, reducing the amount of target antioxidants injected by diluting the sample is the primary method to avoid saturation of the sample signal. Zhou et al. found that controlling sample injection not only avoided signal saturation but also reduced sample residue in the study of metabolomics analysis by chemical isotope labeling LC-TOF-MS [42].

The signal response and quantitative results are controlled within the experimental threshold by sample dilution when analyzing a single compound or multiple compounds of comparable content in the sample. However, the feasibility of diluting the sample is poor in simultaneous quantitative detection of multiple compounds with large differences in content. This can result in low-level compounds not being detected.

When a compound contains non-single isotopes, its molecular ion produces isotope peaks. In general, the lightest isotope has the highest natural abundance, and the molecular ion peak [M]^+^ is also produced by the lightest isotope. In mass spectrometry, the main peaks of molecular ions and fragment ions are generally composed of the most abundant isotopes in nature and also the lightest isotope of each element, including ^1^H, ^12^C, ^14^N, and ^16^O. In the absence of M + H and with less interference from impurities, the chromatographic peak (M + 1) of the low relative abundance is formed by heavy isotopes with low natural content. Except for Cl and Br atoms, the relative abundance of the isotopic peaks of common atoms such as C, H, O, and N atoms is extremely weak. Quantitative analysis of sinapine can be performed with its isotope ion peaks because there are only C, H, O, and N in sinapine (C_16_H_24_NO_5_).

**Figure 1:**
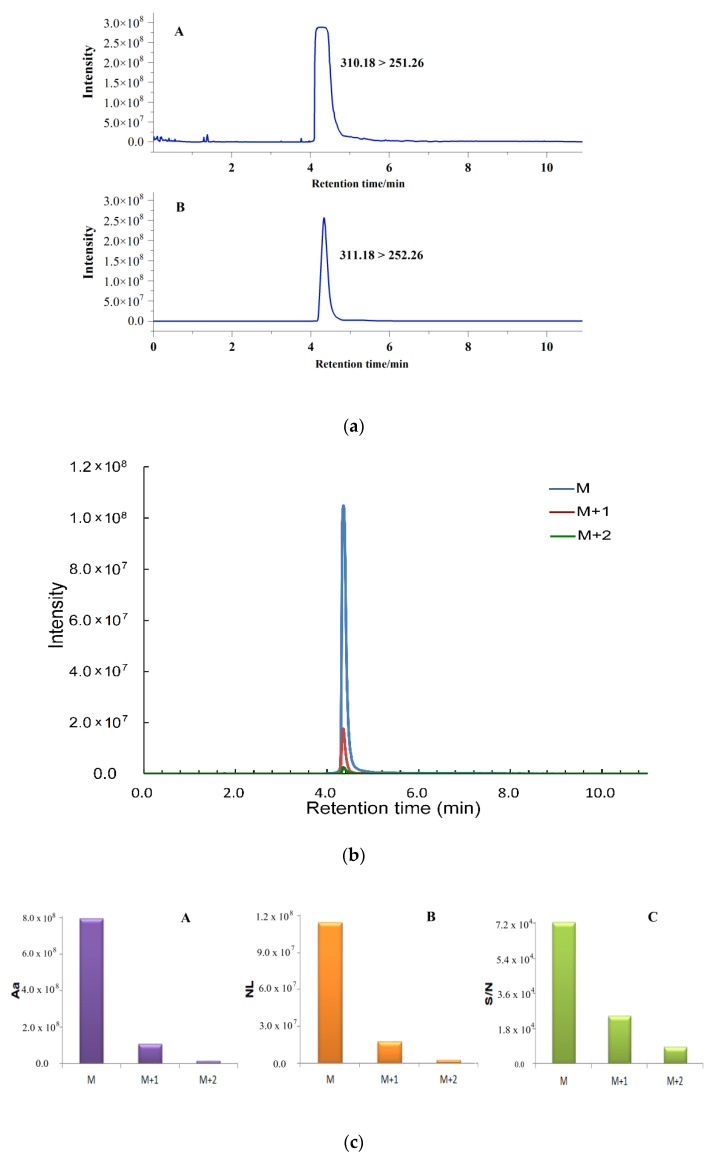
(**a**) Chromatogram of molecular ion (A: [M]^+^) and isotope ion (B: [M + 1]^+^) of sinapine in rapeseed; (**b**) chromatogram of isotopic ions of sinapine in standard solution; and (**c**) comparison of peak area, response value and signal-to-noise ratio of isotope ion chromatogram of sinapine (5 mg/kg).

Figure 1a shows the chromatograms obtained with [M]^+^ and [M + 1]^+^, respectively. There was a clear signal saturation phenomenon in Figure 1a-A and accurate quantification analysis could not be achieved because the chromatographic peak shape is not standard. Figure 1a-B shows that the isotope peak shape of the sinapine was suitable, the peak time and the baseline were stable, and there was no signal saturation phenomenon. Thus, this approach can be used for quantitative analysis of sinapine. According to the secondary mass spectrum of the isotope peak ([M + 1]^+^), the fragment ion of 252.26 was selected for accurate quantification in this experiment.

### 2.2. Simultaneous Quantification by Isotope Ion and Product Ions

This method could significantly reduce the signal response of high-content compounds. Theoretically, the abundance ratio of the isotope ion peak and the main peak of the molecular ion peak is equal to the ratio of the heavy isotope to the light isotope in each element multiplied by the number of atoms of the element in the molecule. The carbon, hydrogen, oxygen, and nitrogen in sinapine have multiple isotopes, which leads to the formation of a variety of sinapine molecules. Therefore, it is difficult to accurately calculate the abundance ratio of the molecular ion peak and the isotope ion peak of sinapine according to theoretical knowledge. It is necessary to explore the difference between the peaks obtained by [M]^+^, [M + 1]^+^, and [M + 2]^+^ as quantitative ions by specific experiments. Figure 1b shows a chromatogram of the molecular ion peak and the isotope ion peak of sinapine detected under the same experimental conditions. Compared with the molecular ion peak, the intensity response and peak area of the isotope ion peak are greatly reduced, the peak shape is suitable, and the retention time is stable. The result indicates that [M + 1]^+^ or [M + 2]^+^ and the corresponding isotope fragment ions can be selected for quantitative analysis of high levels of compounds, such as sinapine. This facilitates the simultaneous analysis of micro-components and trace components.

The peak area (Aa), nominal level (NL), and signal-to-noise ratio (S/N) of the isotope peaks of the sinapine standard solution at different concentrations (1 mg/kg, 5 mg/kg, and 8 mg/kg) were determined in this study (Figure 1c). The data showed that the peak area ratio of [M]^+^ peak to [M + 1]^+^ peak of sinapine at three different concentrations was about 7 (6.99–7.35), and the nominal level ratio was also about 7 (6.51–6.88). In addition, the signal-to-noise ratio of the molecular peak ([M]^+^) is about three times that of the isotopic peak ([M + 1]^+^, 2.89–2.96). The ratios of Aa, NL, and S/N between the two isotopic peaks ([M + 1]^+^ and [M + 2]^+^) of sinapine at three different concentrations were similar. The isotopic ion peak ([M + 1]^+^) was selected for quantitative analysis of sinapine, collectively considering the S/N, Aa, and NL of the chromatographic peak.

**Figure 2:**
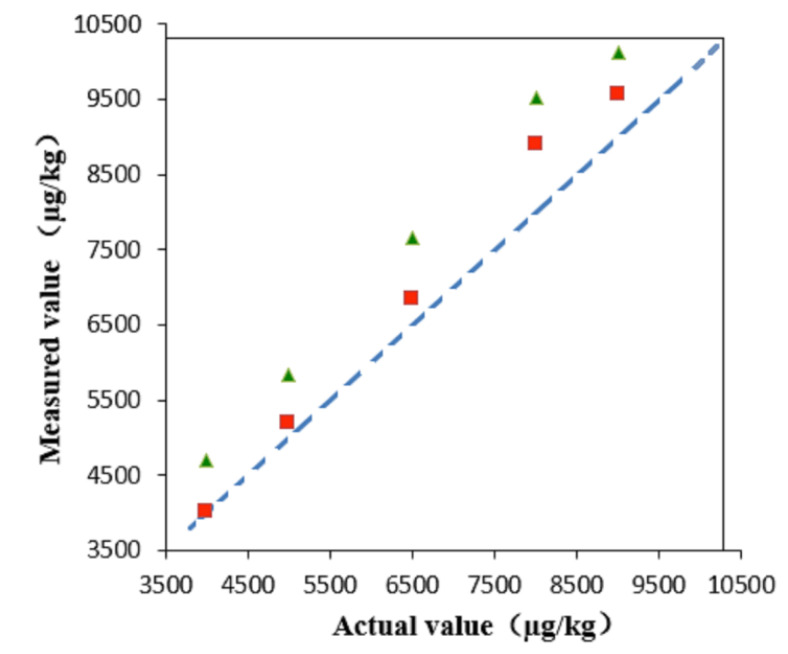
Comparison of quantitative results of sinapine by molecular ion (green triangles) and isotopic ion (red squares).

This method could also improve the detection accuracy of high-content compounds. The quantitative analysis by using the sinapine molecular ion peak and isotope ion peak is shown in Figure 2. The isotope ion peak ([M + 1]^+^) was closer to the actual value, and the experimental error was smaller. This may be because the sinapine signal near the saturation state causes a decrease in signal sensitivity, which ultimately results in significant quantitative accuracy reduction when the molecular ion peak is used for quantitation. This indicates that quantification via the isotope ion peaks can improve the detection accuracy of high-level compounds when the compound signal is saturated.

**Figure 3:**
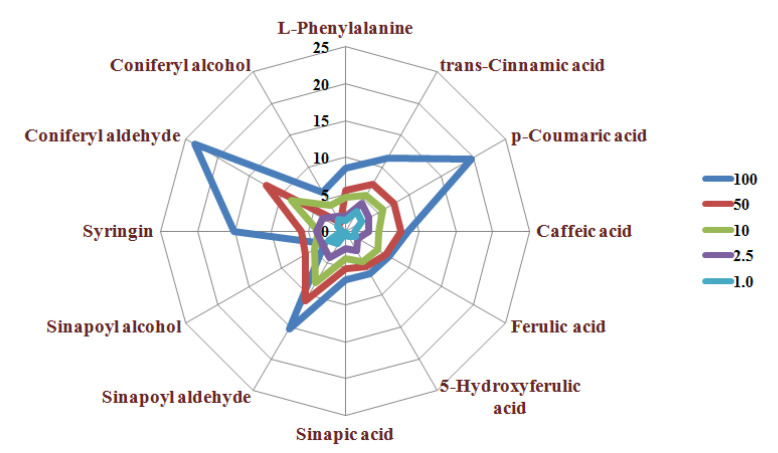
Repeatability of quantitative results of target compounds detected at different dilution ratios.

In addition, this method could improve the stability and repeatability of low-content components. Sample dilution has a large impact on the detection of low-level compounds. The repeatabilities (RSD%) of the measured values of the phenolic compounds at different dilution factors are shown in Figure 3. Samples near the center point of the radar graph have a smaller RSD% of the measured value (*n* = 3) and, thus, better repeatability and stability. The RSD% of the polyphenol compound measurement results differed greatly at different dilution factors (Figure 3). The RSD% of the polyphenol compound measurement value was the largest when the rapeseed sample extract was diluted 100 times. The RSD% of the measured value of the target compounds gradually decreased as the dilution factor decreased. This is mainly because the signal intensity of the target compound is relatively weak after dilution and is more susceptible to interference from the background signal and the sample matrix. This ultimately leads to reduced repeatability and stability. Conversely, the signal of the target analyte will be enhanced to reduce the interference of other signals when the dilution factor is reduced. The stable signal will lead to better repeatability. A smaller dilution factor implies better stability and repeatability of the measured values, but this can cause signal saturation and cannot be quantified. However, the use of isotope peaks for quantification not only avoids signal saturation of high-level compounds such as micro-components to improve the detection accuracy but can also improve the stability and repeatability of low-level compounds, such as trace components, by appropriately reducing the dilution factor. Our approach considers the problem of mass spectrometry residue caused by high concentrations of samples and standards; the rapeseed samples were finally diluted 50 times for simultaneous quantitative analysis of polyphenols in this study.

### 2.3. Method Validation

#### 2.3.1. Linear Range, Limit of Detection, and Limit of Quantitation

Different concentrations of the mixed standard solution were accurately prepared and analyzed according to optimized liquid phase and mass spectrometry conditions. To investigate the linear range of the method, standard curves were established with a seven-point method based on the peak area (y) and concentration (x, μg/L) of the target antioxidants at different concentrations. The limits of detection (LODs) and limits of quality (LOQs) of the polyphenolic compounds were determined at a signal-to-noise ratio (S/N) of 3 and 10, respectively. The regression linear range, as well as the LODs and LOQs of each target, are shown in Table 1. The target compounds have a wide linear range and suitable linear relationship (0.9970 ≤ determination coefficient (R^2^) ≤ 0.9997). The low LODs and LOQs for target polyphenols were 0.01–9.84 μg/kg and 0.03–32.80 μg/kg, respectively. The linearity, LODs, and LOQs indicated that our proposed simultaneous quantitative detection method has high sensitivity and wide applicability.

#### 2.3.2. Precision, Recovery, and Matrix Effect

Precision refers to the degree of coincidence between the repeated measurements of the sample. This is usually expressed as relative standard deviation (RSD%). Intra-day precision and inter-day precision of the method are generally investigated. Intra-day precisions and inter-day precisions of target phenols were obtained by continuously measuring different spiked quality control samples three times in one day and over five consecutive days, respectively. The intra-day precisions of the method ranged from 1.5% to 8.4%, and the range of inter-day precisions was 3.3–14.4% (Table S3). This suggests that the stability of the proposed quantitative detection method is suitable.

The accuracy is used to assess the degree of coincidence between the measured value and the actual value, expressed in terms of measurement error. However, in fact, the recovery is usually used to evaluate the accuracy of the method since the actual value is unknown. Rapeseed samples with three different spiked levels were pretreated and analyzed in this experiment. Recoveries were obtained by calculating the ratio of the difference between the measured value of the spiked rapeseed samples at three different spiking levels and the measured value of the samples to the spiking levels. Table S3 shows that the recoveries of 13 compounds ranged from 81.9% to 117.2%.

The matrix effect refers to the effect of other components in the sample matrix on the determination accuracy of the targets [43]. The quality control sample extract with three different levels of standard solutions was measured three times to determine the matrix effect. The matrix effects can significantly affect the ionization efficiency of the target compound. Matrix effects of polyphenolic compounds were calculated according to the reported method [44]. Table S3 shows that the matrix effects of all targets were lower than 13.7%, which indicated that matrix effects were not obvious. The influence of matrix effects on the measured value of the phenolic compounds could be ignored. In summary, the simultaneous analysis method could be applied to the quantitative detection of phenolic compounds in rapeseeds because the precision, recovery, and matrix effect of the method were acceptable.

## 3. Method Application

**Figure 4:**
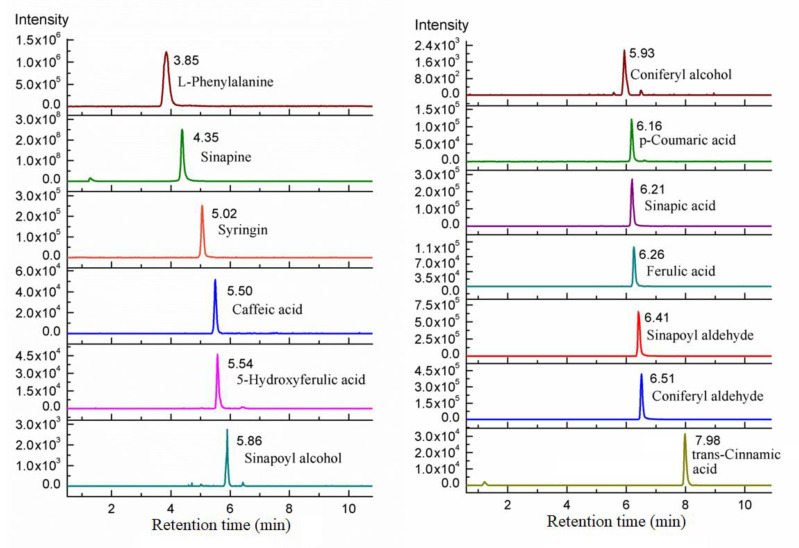
Chromatograms of 13 target compounds in rapeseeds.

The simultaneous quantitative detection method was applied to rapeseed samples to obtain a chromatogram of 13 target compounds (Figure 4). All of the compounds were determined at a corresponding retention time, and the chromatographic peaks achieved baseline separation. The content of phenolic compounds in rapeseed was 84.4 ± 35.1 mg/kg for l-phenylalanine, 4.5 ± 2.5 mg/kg for trans-cinnamic acid, 2.6 ± 1.5 mg/kg for p-coumaric acid, 2.9 ± 0.6 mg/kg for caffeic acid, 7.8 ± 2.6 mg/kg for ferulic acid, 6.8 ± 0.6 mg/kg for 5-hydroxyferic acid, 345.0 ± 111.9 mg/kg for sinapic acid, 8280.5 ± 2144.4 mg/kg for sinapine, 0.4 ± 0.1 mg/kg for sinapoyl aldehyde, 24.2 ± 3.7 mg/kg for sinapoyl alcohol, 0.4 ± 0.2 mg/kg for syringin, 0.2 ± 0.1 mg/kg for coniferyl aldehyde, and 7.7 ± 1.0 mg/kg for coniferyl alcohol. These results are consistent with previous reports [45,46,47].

**Figure 5:**
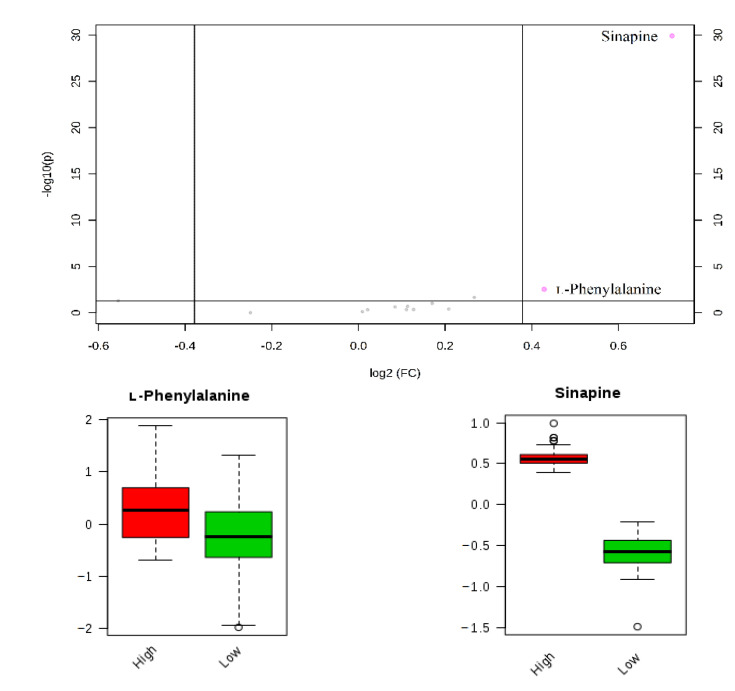
Volcano plot of phenolic compounds and box-plot of differential compounds.

Statistical analysis of phenolic compounds in rapeseed was performed by using the MetaboAnalyst 4.0 online analytical platform. The differential analysis of high and low phenolic rapeseed samples rationally set the values of two important indicators in the volcano plot (*p*-value of 0.05 and fold-change of 1.4). The volcano plot of 13 target compounds was plotted with log_2_ (FC) and −log_10_ (P) as the horizontal coordinate and vertical coordinate, respectively. The volcano plot and box-plot of 13 target compounds in high and low phenolic rapeseed are shown in Figure 5. The results indicated that l-phenylalanine and sinapine were the main differential compounds in the high and low total phenolic rapeseed samples. That is, the difference in the content of sinapine causes a difference in the TPC of rapeseed.

Recent studies have shown that sinapine is a very valuable natural antioxidant with antibacterial, hypolipidemic, liver-protecting, anti-radiation, anti-cancer, and anti-tumor effects [48]. Sinapine is mainly found in rapeseed kernels [49] and is easily hydrolyzed to sinapic acid and then further decarboxylated to form a fat-soluble canolol with high antioxidant activity under high-temperature and high-pressure conditions [50]. Canolol can significantly improve the nutritional quality of rapeseed oil. Research on methods for promoting the conversion of sinapine to canolol in rapeseed is quite active. For example, Li et al. reported a new method for pressurized solvent extraction that can efficiently extract sinapine and efficiently convert it into canolol [51]. Sinapine is expected to become an important active ingredient in the future pharmaceutical market due to its suitable anti-cancer functions. Rapeseed with high sinapine content is also a high-quality raw material for high-nutrient-quality rapeseed oil rich in canolol.

## 4. Methods

### 4.1. Chemicals and Materials

Phenylalanine (chemical purity ≥ 98%) was purchased from Shanghai Aladdin Biotechnology Co., Ltd. (Shanghai, China). Trans-cinnamic acid (chemical purity ≥ 98%), p-coumaric acid (chemical purity ≥ 98%), caffeic acid (chemical purity ≥ 98%), sinapic acid (chemical purity ≥ 98%), sinapoyl aldehyde (chemical purity ≥ 95%), and syringin (chemical purity ≥ 98%) were purchased from Shanghai Yuanye Biotechnology Co., Ltd. (Shanghai, China). Ferulic acid (chemical purity ≥ 99%) was purchased from Beijing Bellingway Technology Co., Ltd. (Beijing, China). The 5-hydroxyferulic acid (chemical purity ≥ 98%) was purchased from Shanghai Zhenzhun Biotechnology Co., Ltd. (Shanghai, China). Sinapine (chemical purity ≥ 99%) was purchased from Dessite Biotechnology Co., Ltd. (Chengdu, China). Sinapoyl alcohol (chemical purity ≥ 98%) was purchased from Koryuan Biomedical Co., Ltd. (Wuhan, China). Coniferyl aldehyde (chemical purity ≥ 98%) and acetic acid (chemical purity ≥ 99.8%) were purchased from Sigma-Aldrich Co., Ltd. (Shanghai, China). Coniferyl alcohol (chemical purity ≥ 98%) was purchased from Alfa Aesar Chemical Co., Ltd. (Shanghai, China). Methanol (chemical purity ≥ 99.9%) was purchased from Thermo Fisher Scientific Co., Ltd. (Shanghai, China). Here, 30 low total phenol rapeseed samples and 30 high total phenol rapeseed samples from different regions were selected. Rapeseed samples were ground with an 80350-CN grinder (Hamilton Beach Electric Co., Ltd., Shenzhen, China) and stored in a BCD-260WDBD refrigerator (Haier Co., Ltd., Qingdao, China) at −4 °C for use.

### 4.2. Preparation of Standard Solution

The l-phenylalanine, trans-cinnamic acid, syringin, p-coumaric acid, sinapic acid, caffeic acid, sinapine, 5-hydroxyferic acid, ferulic acid, sinapyl alcohol, coniferyl alcohol, and other standards were weighted to 5.0 mg with a CPA224S electronic analytical balance (Sartorius, Göttingen, Germany). They were then dissolved in methanol, and the volume was made up to 10.0 mL. Different single working standard solutions of 500 mg/L were obtained, stored in a brown bottle without loss, and stored in a refrigerator at −20 °C. The series of dilutions were made in the concentration range 100–12,000 mg/L for sinapine and 0.1–800 μg/L for trace phenolic compounds. These standard solutions were stored in a −4 °C refrigerator before further use.

### 4.3. Sample Preparation

The 0.1 g of rapeseed powder was weighed into a 5 mL stoppered centrifuge tube (Agilent Technologies Co., Ltd., Shanghai, China) followed by 1 mL of 50% methanol solution. This was then fully mixed on the XH-C vortex mixer (Changzhou Future Instrument Manufacturing Co., Ltd., Changzhou, China) and shaken for 1 h at room temperature in the dark with a KB-5010 Shaker (Linbeier Instrument Manufacturing Co., Ltd., Haimen, China). The samples were then centrifuged at 4500 r/min for 8 min in a HITACHI CT6E centrifuge (HITACHI, Tokyo, Japan). The supernatant was quantitatively transferred to another 5 mL stoppered centrifuge tube. Next, 1 mL of 50% methanol solution was added to the lower layer of the precipitate, and the precipitate was spun on a vortex mixer, shaken for 1 h, and centrifuged at 4500 r/min for 8 min. Each rapeseed sample was repeatedly extracted three times. Finally, all of the extracts (3.0 mL total) were mixed and filtered through a 0.22 μm organic membrane (American Millipore Company, Beijing, China). Next, 20 μL of the filtrate was accurately removed and diluted with methanol to 1 mL for analysis by LC-MS/MS.

### 4.4. LC-MS/MS Analysis

The LC-MS/MS system includes a triple quadrupole mass spectrometer Thermo TSQ Quantum Ultra EMR (Thermo Fisher Scientific, Waltham, MA, USA) and an Accela HPLC liquid phase system (Thermo Fisher Scientific, Waltham, MA, USA). The liquid chromatographic conditions were as follows: The column was a Thermo Syncronils C18 reversed-phase column (100 × 2.1 mm, 3 μm) (Thermo Fisher Scientific, Waltham, MA, USA). Due to the polarity of most phenolic compounds, the weakly acidic alcohol-water system has a strong ability to elute phenolic compounds. Mobile phase A was 0.02% acetic acid in water (*v*/*v*), and mobile phase B was 0.02% acetic acid in methanol (*v*/*v*). The flow rate of the mobile phase was set to 200 μL/min, and the injection volume was 5 μL; the column temperature was maintained at around 30 °C with a detailed gradient elution procedure shown in Table S1.

Mass Spectrometry Conditions: MS/MS data acquisition was performed on a Thermo TSQ Quantum MS triple quadrupole mass spectrometer equipped with an electrospray ionization interface. Heatable Electron Spray Ionization (ESI) ion source and Selective Reaction Monitoring (SRM) scan mode were selected. Negative or positive ion scanning was selected depending on the characteristics of the target compound. The optimum needle position was adjusted based on the liquid flow rate and signal response intensity; the final probe position was adjusted to the C position. Detailed ion source parameters include spray voltage, vaporizer temperature, and auxiliary gas pressure (Table S2).

Flow injection was used to obtain accurate ion fragmentation information for different compounds. A positive ion ([M]^+^, [M + H]^+^, [M + Na]^+^) or a negative ion mode ([M − H]^−^) was selected for Q_1_ scanning according to the chemical properties of the target. Other parameters such as the flow rate of the peristaltic pump, gas flow, voltage, and temperature of the ion source were adjusted such that the signal intensity was greater than 10^6^and maintained at a steady state. A parent ion was collided in the Q_2_ quadrupole and screened in the Q_3_ quadrupole. Finally, the target number of fragment ion information was obtained and sorted according to the relative abundance. Optimized mass spectrometry scan parameters, such as collision energy and tube lens value, are shown in Table 2.

### 4.5. Data Processing and Analysis

Qualitative analysis of targets in the rapeseed sample was performed by comparing the retention time and the mass spectrum of the target phenolic compound standard under the same liquid phase and mass spectrometry conditions. Here, an external standard method was used for accurate quantitative determination. The final analytical concentration of the standard solution was plotted on the abscissa, and the peak area of the chromatogram was taken as the ordinate. The standard curves were established by a seven-point method. Batch sample data were processed via Xcalibur Version 2.0.7 software (Thermo Fisher Scientific, Waltham, MA, USA). Statistical analysis and charting were achieved primarily through online MetaboAnalyst 4.0. 

## 5. Conclusions

A new quantification strategy was proposed by using product and isotope ions to detect trace and micro phenolic compounds together. Herein, 13 polyphenols in the phenylpropanoid biosynthesis pathway were also achieved via this method to resolve challenges in the simultaneous quantitative determination of micro and trace components. The use of isotope ion for quantification not only reduces the signal response to solve the problem of sinapine signal saturation in rapeseed but also improve the detection accuracy, stability, and repeatability of the method. The differences in the peak area, nominal level, and signal-to-noise ratio between the molecular ion peak and the isotope ion peak of sinapine were also investigated.

This LC-MS/MS method has suitable stability, high sensitivity, acceptable recoveries, and low matrix effects. The application of this method in rapeseed showed that there was a significant difference in the content of l-phenylalanine and sinapine in the phenylpropanoid biosynthesis pathway of the low and high TPC rapeseeds. Sinapine is an antioxidant and is also a precursor of canolol, which greatly enhances the nutritional quality of rapeseed oil during processing. The method provides a basis for detection technology for the screening of high phenolic rapeseed germplasm resources and the regulation mechanism of polyphenol formation in rapeseed to further promote sustainable and healthy development in the rapeseed industry. This approach also provides a new and feasible strategy for the simultaneous quantitative analysis of other micro- and trace components.

## Figures and Tables

**Table 1:** Linear range, regression equation, determination coefficient (R^2^), LODs, and LOQs of antioxidants.

Analytes	Linear Range (μg/kg)	Regression Equation	R^2^ Value	LOD/(μg/kg)	LOQ/(μg/kg)
l-Phenylalanine	0.5–800	y = 91,196.0x + 147,656.0	0.9995	0.05	0.16
trans-Cinnamic Acid	0.1–500	y = 1854.1x − 1514.7	0.9996	0.01	0.04
p-Coumaric Acid	0.1–500	y = 8764.9x + 11,452.1	0.9995	0.03	0.11
Caffeic Acid	0.1–800	y = 3485.9x − 2347.2	0.9997	0.08	0.25
Ferulic Acid	0.1–800	y = 5993.3x + 21,684.8	0.9996	0.01	0.03
Coniferyl Aldehyde	0.1–400	y = 24,408.9x + 95,830.1	0.9986	0.02	0.06
Coniferyl Alcohol	0.5–500	y = 100.9x − 451.2	0.9991	0.09	0.31
5-Hydroxyferulic Acid	0.5–600	y = 3088.2x − 15,869.9	0.9986	0.13	0.42
Sinapic Acid	0.1–800	y = 5299.4x − 3167.6	0.9996	0.01	0.04
Sinapoyl Aldehyde	0.1–400	y = 33,627.5x + 33,970.9	0.9987	0.02	0.06
Sinapoyl Alcohol	0.5–800	y = 64.1x − 954.8	0.9973	0.15	0.49
Syringin	0.1–500	y = 16,670.9x + 40,916.1	0.9994	0.02	0.07
Sinapine	100–12,000	y = 12,734.2x + 12,326.0	0.9970	9.84	32.80

**Table 2:** Retention time and scan parameters of target compounds.

Targets	Retention Time/(min)	Parent Ion/(*m*/*z*)	Product Ion /(*m*/*z*)	Collision Energy/(eV)	Ion Polarity	Tube Lens/(V)
l-Phenylalanine	3.85	166.076	120.300 > 103.300	12/25	[M + H]^+^	96
trans-Cinnamic Acid	7.98	147.050	103.350	14	[M − H]^−^	88
p-Coumaric Acid	6.16	163.047	119.247	16	[M − H]^−^	50
Caffeic Acid	5.50	179.042	135.282	16	[M − H]^−^	60
Ferulic Acid	6.26	193.020	134.020 > 178.021	18/16	[M − H]^−^	57
Coniferyl Aldehyde	6.51	177.060	162.210	14	[M − H]^−^	88
Coniferyl Alcohol	5.93	179.070	146.070 > 164.050	16/18	[M − H]^−^	72
5-Hydroxyferulic Acid	5.54	209.051	150.170 > 194.150	18/13	[M − H]^−^	90
Sinapic Acid	6.21	223.050	208.160 > 193.090	12/22	[M − H]^−^	86
Sinapoyl Aldehyde	6.41	207.060	177.160 > 192.180	21/16	[M − H]^−^	95
Sinapoyl Alcohol	5.86	209.089	179.089	23	[M − H]^−^	64
Syringin	5.02	395.200	232.200	22	[M + Na]^+^	120
Sinapine	4.35	311.180	252.260	15	[M + 1]^+^	78

## Data Availability

The data presented in this study are available upon reasonable request from the corresponding author. Data are not publicly available due to the terms of the ethical approval.

## Supplementary Materials

The following are available online at https://www.mdpi.com/article/10.3390/metabo11090589/s1, Table S1: The gradient elution program, Table S2: Parameters of the ion source, Table S3: Precisions, recoveries, matrix effects of targets.
