# Supplementary material for: Simultaneous Quantification of Trace and Micro Phenolic Compounds by Liquid Chromatography Tandem-Mass Spectrometry
Source: Metabolites. 2021 Aug 31;11(9):589. doi: 10.3390/metabo11090589 (PMC8467048; doi:10.3390/metabo11090589)
Supplement: Supplementary file 1 [file metabolites-11-00589-s001.zip › metabolites-1341759-supplementary.pdf]

## Supplementary Materials

Table S1 The gradient elution program

Table S2 Parameters of the ion source

Table S3 Precisions, recoveries, matrix effects of targets

Table S1 The gradient elution program

| Time（min） | Mobile phase A | Mobile phase B |
|---|---|---|
| 0.00 | 90 | 10 |
| 1.00 | 90 | 10 |
| 2.50 | 55 | 45 |
| 5.00 | 30 | 70 |
| 7.50 | 30 | 70 |
| 8.50 | 20 | 80 |
| 8.60 | 10 | 90 |
| 11.00 | 10 | 90 |

Table S2 Parameters of the ion source

| Parameters | Positive ion mode | Negative ion mode |
|---|---|---|
| Spray Voltage (V) | +4000 | -3000 |
| Capillary Temperature (°C) | 320 | 320 |
| Vaporizer Temperature (°C) | 280 | 280 |
| Auxiliary Gas ($N_2$) | 15 | 15 |
| Sheath Gas ($N_2$) | 40 | 40 |
| Collision gas (Ar) | 1.5 m Torr | 1.5 m Torr |
| Cycle Time (s) | 0.7 | 0.7 |
| Q1 Peak Width (FWHM, Da) | 0.7 | 0.7 |

Table S3 Precisions, recoveries and matrix effects of targets

| Analytes | Spiked Level /(μg/kg) | Intra-day Precision /(RSD %, n = 3) | Iner-day Precision /(RSD%, n=5) | Recovery /(%, n=3) | Matrix Effect /(%, n=3) |
|---|---|---|---|---|---|
| L-Phenylalanine | 1/50/200 | 3.4/1.6/4.4 | 6.8/3.3/7.2 | 104.2/99.1/103.6 | 8.6/5.4/6.7 |
| trans-Cinnamic acid | 1/50/200 | 2.8/1.5/3.3 | 4.9/6.1/8.0 | 82.1/102.6/94.3 | -10.2/-6.4/-8.8 |
| p-Coumaric acid | 1/50/200 | 8.4/4.3/2.8 | 9.2/3.8/5.9 | 95.7/99.4/105.0 | -4.3/-3.9/-4.3 |
| Caffeic acid | 1/50/200 | 6.7/5.8/6.9 | 9.3/7.6/8.5 | 108.5/103.4/109.1 | 11.1/7.5/10.4 |
| Ferulic acid | 1/50/200 | 5.9/6.4/4.4 | 8.9/7.2/3.9 | 81.9/94.0/90.6 | -11.5/-6.4/-9.3 |
| Coniferyl aldehyde | 1/50/200 | 7.9/5.4/8.0 | 11.7/6.0/9.1 | 90.4/95.1/89.2 | -7.2/-6.3/-10.4 |
| Coniferyl alcohol | 1/50/200 | 5.0/3.3/5.8 | 8.3/5.2/9.4 | 109.7/101.6/108.3 | 9.6/7.8/7.4 |
| 5-Hydroxyferulic acid | 1/50/200 | 7.6/4.7/5.1 | 14.4/6.9/10.8 | 106.4/97.1/103.7 | 9.9/8.2/7.8 |
| Sinapic acid | 50/200/500 | 5.1/2.6/4.2 | 8.4/5.2/7.9 | 90.5/103.1/97.4 | -8.7/-5.4/-6.4 |
| Sinapoyl aldehyde | 1/50/200 | 7.3/3.0/7.6 | 8.2/6.5/13.1 | 94.7/88.9/86.1 | -6.1/-7.7/-9.5 |
| Sinapoyl alcohol | 1/50 /200 | 4.2/2.6/6.3 | 6.7/8.8/9.6 | 101.4/97.6/94.2 | 8.7/6.8/5.1 |
| Syringin | 1/50/200 | 2.0/4.9/7.8 | 9.8/4.1/5.9 | 86.5/90.7/85.1 | -9.4/-5.2/-9.0 |
| Sinapine | 2000/3000/5000 | 6.5/3.4/2.5 | 9.3/7.8/9.1 | 117.2/105.4/103.5 | 13.7/6.7/6.4 |
